# Evaluating the impact of lipids in isolated islet research

**DOI:** 10.3389/fendo.2025.1548596

**Published:** 2025-07-02

**Authors:** Emelien M. Jentz, Jamie W. Joseph

**Affiliations:** School of Pharmacy, University of Waterloo, Kitchener, ON, Canada

**Keywords:** islets, insulin secretion, free fatty acids, albumin, diabetes

## Abstract

Pancreatic β-cells secrete insulin in response to nutrient stimulation. Glucose, amino acids and free fatty acids (FFA) have all been shown to stimulate insulin release *in vivo*. *In vitro*, isolated islet studies have also demonstrated similar results to those seen *in vivo*. It has also been shown that high-fat diet-fed mice or chronic treatment of isolated islets to high glucose and FFA can lead to glucolipotoxicity and impaired β-cell function. Isolated islet studies are a standard assay for preliminary testing of novel ideas and drugs related to islet function. Interpreting and comparing *in vitro* islet results from acute and chronic treatment of nutrients can be difficult since a wide variety of methods are used to isolate and culture islets and assess islet function. In this review, we compare *in vivo* and *in vitro* FFA absorption, transport and metabolism and discuss *in vitro* methods and concepts related to islet responses to nutrients, focusing on the effects of fatty acids on insulin secretion and β-cell function. This review also discusses FFA levels and transport seen in type 2 diabetes and compares them to how isolated islets are treated with FFA *in vitro*.

## Introduction

Type 2 diabetes occurs when the pancreas does not secrete enough insulin, and the body’s cells do not effectively respond to the insulin secreted. In order for glucose to stimulate insulin secretion, it must be metabolized by the pancreatic β-cell. Glucose enters the β-cell through glucose transporter 2 (GLUT2) in rodents and GLUT1 and GLUT3 in humans and is metabolized by glycolysis and the tricarboxylic acid cycle, leading to the production of adenosine triphosphate (ATP) via oxidative phosphorylation (1–5). The increased ATP causes a rise in the ATP/ADP ratio, leading to the closure of ATP-sensitive K^+^-channels and plasma membrane depolarization (6). This depolarization opens the voltage-dependent Ca^2+^ channels, resulting in an influx of calcium. A high concentration of calcium leads to the exocytosis of insulin-containing granules (7). In addition to the above-described triggering pathway of insulin secretion, there is an amplifying pathway involving other metabolites. The mechanisms by which these metabolites potentiate insulin secretion are still under debate (2–5).

In addition to glucose, lipids and amino acids can also regulate insulin release from β-cells. It has been shown that acute stimulation with exogenous non-esterified fatty acids (NEFA) or free fatty acids (FFA) potentiates glucose-stimulated insulin secretion (GSIS) (8, 9). Most of the free unbound forms of these fatty acids have been shown to potentiate GSIS; however, palmitic acid is the most potent (10). In contrast, chronic stimulation with exogenous FFA reduces GSIS (11, 12). Inside pancreatic β-cells, the esterification of FFAs to long-chain acyl-CoA derivative of the FFA is a key step, and they can have direct and indirect effects on insulin secretion (13). There is increasing interest in understanding the role of FFA in the development of β-cell dysfunction in type 2 diabetes, and isolated islets are a key early model to assess the effects of FFA.

One difficulty in performing FFA studies in islets is interpreting and comparing experiments between research groups. Looking at the effects of lipids on β-cell function is complicated since there are numerous types of fatty acids, each with a unique chain length and degree of saturation. Also, how the FFA are prepared and conjugated to albumin can make interpreting and comparing results from different studies difficult. In this review, we will discuss FFA solubility and transport in the blood and the changes in FFA seen in type 2 diabetes and compare this to how lipid studies are done in isolated islets. This review will also discuss some of the methodological variability in the literature that makes comparing studies difficult and make suggestions for future islet studies.

## Solubility of plasma FFA

**Figure 1:**
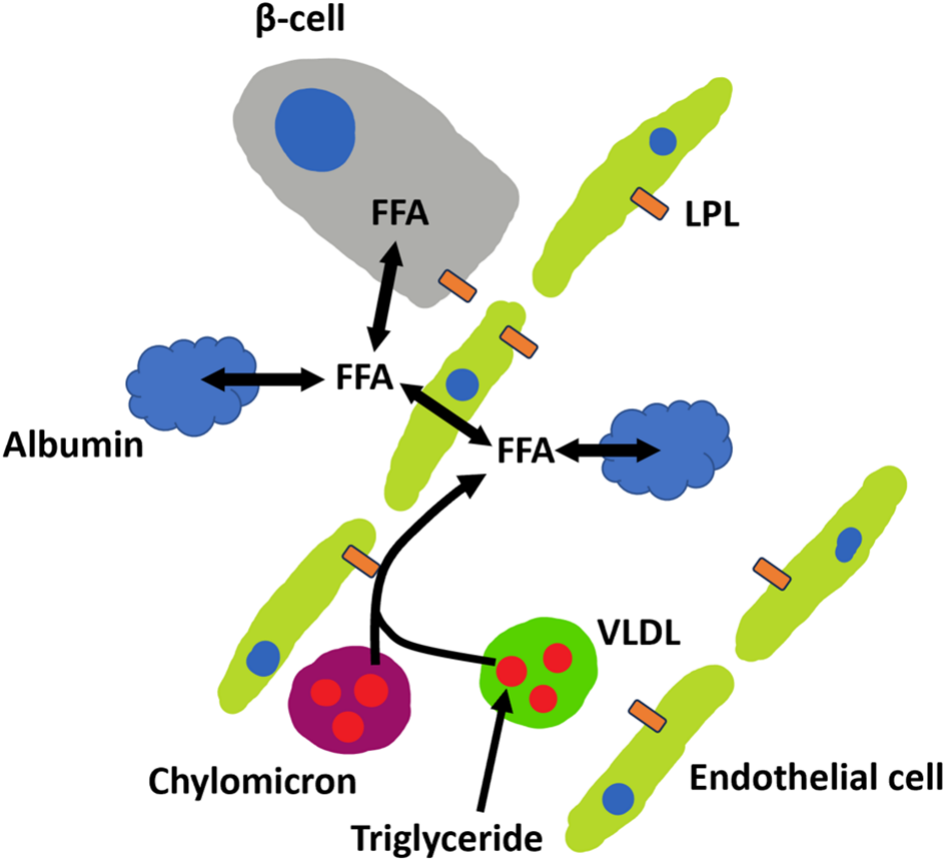
Transport of plasma FFA to islet β-cells *in vivo*. Chylomicrons (purple), VLDL (green) and albumin (blue) can all deliver FFA to β-cells. Albumin controls free unbound FFA found in circulation. Unbound FFA can be transported through the blood vessel wall into the interstitial space, where it can bind to interstitial albumin or enter β-cells. FFA can also be released from triglycerides (red) found in chylomicron and VLDL particles by lipoprotein lipase (LPL) found on endothelial cells (green).

The five main plasma FFAs (palmitate, oleate, stearate, linoleic acid and arachidonic acid) are part of a group of FFA, also referred to as Long-chain FFAs, that have a carbon length of 13–20 C. Long-chain FFAs are only water-soluble at very high pH values (>10) (14–16). The concentration of FFAs in an aqueous solution is difficult to measure and can only be measured indirectly (17). The solubility is also dependent on pH, temperature and type of fat. For example, without any stabilizers such as albumin, palmitate solubility is between 4-16 µmol/L (18, 19). Because of the low solubility, most of the triglycerides (TG), phospholipids and cholesterol in blood plasma are carried by lipoproteins, whereas most of the FFAs are bound to albumin, and only a small fraction of circulating lipids are unbound to a carrier protein. FFAs are transported primarily by albumin to increase the solubility in blood plasma (20). Albumin is required to stabilize FFAs in blood and the interstitial compartment (**Figure 1**) (21, 22). Albumin can bind up to 7 FFAs per molecule, but typically, human albumin has, on average, 2 FFAs bound in human plasma (23). Of the 7 FFA binding sites of albumin, three are high affinity binding sites, and albumin is an effective transporter of FFA as long as its concentration is above 0.5 μM (24). In healthy human plasma, the molar ratio of FFAs to albumin ranges from 0.7:1 to 3:1 and can rise to 6:1 in some rare pathological conditions (25, 26). The transport and delivery of FFAs to cells depend on the FFA/albumin interaction in both plasma and the interstitial compartment between tissue cells (21, 22).

## Bound *vs*. unbound FFA

When blood plasma FFAs (or NEFAs) are measured in humans, they represent unbound and bound FFA. The total unbound FFA concentrations in human plasma are estimated to be between 5 nM and 50 nM; however, the concentration of unbound fatty acids near β-cells is unknown (27, 28). Most *in vitro* islet studies use bovine serum albumin (BSA) for insulin secretion assays to improve solubility and control unbound fatty acid concentrations when FFA is used as a stimulus. Commercial BSA may contain FFAs, and the concentration of individual fatty species is likely different in preparations for BSA (29). In addition, purified albumin may contain variable amounts of other contaminants, which can affect FFA binding and the results of any experiments being performed (29). FFAs are often removed from the BSA before conjugating with a predetermined concentration of FFAs to control the exact lipid composition for treating cells (11). FFAs are commonly conjugated to FFA-free BSA prepared by a company such as SIGMA or charcoal-treated BSA for islet experiments. Charcoal treatment of BSA allows the removal of some contaminants, including lipids (29–34). FFA-free albumins that can be purchased commercially typically have a higher purity. Commercially purchased FFA-free BSA also has less residual fatty acids than charcoal-absorbed BSA (29–34).

The unbound FFA concentrations can be calculated using a multiple-stepwise equilibrium model (21, 30), or they can be measured using a fluorescent probe ADIFAB2, which is an acrylodan-derivatized intestinal fatty acid binding protein (24, 35–38). Calculating unbound FFAs is not always the best way to go since the method used to complex FFAs to albumin can also affect unbound FFAs. For example, it has been shown that FFAs used in the presence of 1% charcoal-absorbed BSA, 0.75% commercial FFA-free BSA or pre-complexed FFAs used in the presence of 0.67% FFA-free BSA result in similar measured unbound FFA concentrations even though the BSA concentration was different between these preparations (36). Albumin also has different affinities for FFAs that depend on the carbon length and degrees of saturation. Palmitate, oleate, stearate and arachidonate all have different affinities for albumin, which will affect unbound FFAs and have an impact on experiments comparing the effects of these lipids (21). It has also been shown that the molar ratio of BSA to FFAs may be used to estimate the final concentration of bound and unbound FFAs. For example, a 0.5 mM sodium palmitate solution with an FFA/albumin molar ratio of 3.3:1 has a theoretical unbound palmitate concentration of 27 nM, and for a similar solution of 0.5 mM sodium oleate with the same molar ratio of FFA/BSA will have an unbound concentration of 47 nM (30). For these reasons and the effects of the method of preparation and batch-to-batch variability, it has been suggested that unbound FFAs should be measured instead of estimated (11). For example, using ADIFAB2, the unbound FFA was about 26 nM for palmitate and 35 nM for oleate in a 1% charcoal-absorbed BSA at an FFA/albumin molar ratio of 3.3:1 which is different from the estimated unbound FFAs for these lipids (36).

## Human fasting plasma lipids

What concentrations of FFAs should be used in *in vitro* experiments with islet β-cells? The answer to this question is not an easy one to determine. For example, when non-fasted or short-term fasting total FFA (e.g., a morning fast) is measured using standard clinical procedures, a typical concentration of about 475 μmol/L in human blood plasma is found (39). Fasting for longer durations can have an impact on the total plasma FFA concentration, rising to about 1000 μmol/L after 24 hours and 2000 μmol/L after 48 hours (40, 41). Since individuals usually only fast overnight before having their first meal of the day, which is followed by another few meals throughout the day, this might suggest that control experiments in islets should be done using a total FFA concentration of around 475 μmol/L. One must also take into consideration the type of fat being used. For example, control islet palmitate experiments should be done at around 109 μmol/L since palmitate accounts for only around 23% of the total FFA concentration in healthy human plasma (39, 42–45). However, most control islet experiments are done without FFA (41, 46–58). It has been shown that circulating plasma FFA are essential for insulin secretion in response to nutrient secretagogues (59, 60). Thus, a lack of FFA in control islet experiments may have its own effects on insulin secretion.

Interestingly, when individual FFA are measured, the non-fasted or short-term fasting total FFA concentrations in healthy human plasma are between 269-5556 μmol/L, with most values falling around 2400 μmol/L, which is only slightly higher than what is measured clinically (43, 44, 61–63). The five main FFA in plasma account for ~85% of the total FFA and include palmitate (16:0, ~23% of total FFA, ~585 μmol/L), oleate (18:1, ~19% of total FFA, ~450 μmol/L), stearate (18:0, ~8% of total FFA, ~159 μmol/L), linoleic acid (18:2, ~31% of total FFA, ~744 μmol/L) and arachidonic acid (20:4, ~8% of total FFA, ~222 μmol/L) (43, 44, 61–63). These studies suggest that standard clinical procedures for measuring the concentration of FFA may slightly underestimate the concentration of FFAs in human plasma. Which concentration of FFA should be used for islet studies? For example, for palmitate, should we use 109 μmol/L calculated from clinical values based on palmitate accounting for ~23% of the total FFA or ~585 μmol/L based on what is measured when individual FFA are measured? A good starting point would be to use a value between these two measurements of ~250-300 μmol/L.

In addition to FFAs, there are other sources of lipids in human plasma, including triglycerides (TG, ~900-1400 μmol/L) which are primarily bound to chylomicrons and very-low density lipoproteins (VLDL), total cholesterol (~3700-5900 μmol/L) with most of the cholesterol bound to high-density lipoproteins (HDL)-cholesterol (~1200-1500 μmol/L) and low-density lipoproteins (LDL)-cholesterol (~2300-3800 μmol/L) (43, 44, 63–68). All of these sources of lipids can be used in the body to form essential cellular structures and signaling molecules or used as an energy source, accounting for about 27% of total dietary energy (39). Chylomicrons and VLDLs can also release FFA from TG in a tissue-specific manner, depending on the body’s needs. Mice are an important animal model for human obesity and diabetes, and their concentration of plasma lipids is similar in mice but not the same. For example, average overnight fasted plasma values from control mice from several papers are FFA 531 μmol/L, triglycerides 1004 μmol/L, total cholesterol 3077 μmol/L, very low-density lipoproteins (VLDL)-cholesterol 422 μmol/L, LDL-cholesterol 379 μmol/L, and HDL-cholesterol 1552 μmol/L (69–74). The differences in plasma lipids between mice and humans may be due to different diets or other physiological factors.

## Postprandial lipid dynamics

An important concept in FFA-stimulated insulin secretion from isolated islets is a rise in FFA after a meal. However, does plasma FFA increase after a meal? The switch from the fasted to fed state inhibits lipolysis from adipocytes and leads to reduced plasma lipid metabolites such as glycerol, FFA and acylcarnitine by about 50-70% postprandially (75). However, it is sometimes assumed when performing *in vitro* islet studies that postprandial plasma FFA rises due to gut absorption, but the opposite is true. The drop in postprandial plasma FFA levels is primarily due to the inhibition of adipocyte lipolysis rather than changes in the absorption of FFA directly into the bloodstream (76). Most FFA absorbed in the gastrointestinal tract are packaged as TGs inside of chylomicrons, and any changes in plasma FFA postprandially are likely due to spill-over during transport (40). The single meal response using a mixed test meal designed after a standard Western diet or other meal types typically results in a slow drop in human plasma FFA going from about 500 μmol/L to 200 μmol/L after about 90 minutes postprandial, followed by a slow rise to 800 μmol/L after 360 minutes (65, 77–86). Total plasma TGs, on the other hand, go from about 1000 μmol/L to a peak of 2000 μmol/L after about 180 minutes. The slow drop in FFA after a meal is likely due to the rapid rise in glucose-induced insulin release that stimulates FFA uptake into tissue cells and inhibition of lipolysis in adipose tissue (85, 86). The subsequent elevation of plasma FFA after about 360 minutes postprandially has been suggested to be part of the switch back to a fasting state (85, 86). The five main FFA found in human plasma also showed similar trends to what is seen with the total changes in plasma lipid profiles postprandially (68, 75, 87).

These studies raise questions about how FFA studies are done in isolated islets. Do these plasma FFA levels represent what the islets see postprandially? If these FFA plasma changes are representative of what islets see after a meal, should isolated islet lipid studies be done similarly if FFA levels drop after a meal? For example, do we pretreat islets with a higher FFA level followed by dropping the FFA when stimulated with high glucose? Another consideration is that since the changes in lipid profiles after a meal showed a slow response, taking hours instead of minutes, should islet lipid studies be performed similarly? Although these questions should be addressed, changes in plasma FFA are unlikely to represent what islets see postprandially. It is important to point out that plasma FFA levels discussed above are for average plasma FFA levels and do not consider local islet lipoprotein lipase release of FFA from other sources such as chylomicrons and VLDL and do not consider changes in local capillary blood flow. Overall, although FFA plays a critical role in regulating *in vivo* islet function, islet exposure and the source of FFA postprandially are unknown.

## Plasma lipid profiles in type 2 diabetes

Type 2 diabetes is a complex metabolic disease that does not have just one underlying cause and involves both genetic and environmental factors. One of the strongest risk factors for type 2 diabetes is obesity (88–90). However, not every obese person gets type 2 diabetes, suggesting other factors must be involved. Changes in diet and plasma FFA are essential players in the obesity-linked development of type 2 diabetes; however, the evidence supporting this link can sometimes be unclear. Several studies, including systematic reviews and meta-analyses, have shown less than strong evidence supporting a possible link between plasma FFA and type 2 diabetes (90–97). The problem with earlier studies is that they focused on total dietary and plasma lipids, which may mask any real effect due to individual lipids that may be involved in the development of type 2 diabetes (40, 76, 98). However, a number of more recent metabolomics studies have not provided any more clarity (43–45, 61–63, 99, 100).

What are the changes in the five main FFAs in type 2 diabetes (T2D) patients? When non-fasted or short-term fasted individual blood lipids are measured, and total FFAs are calculated, it gives values between 269-5556 μmol/L in control patients, whereas in T2D patients, it was between 621-9582 μmol/L (43, 44, 61–63). The five main FFA in plasma accounted for ~85% of matched control patients, and ~86% of T2D patients total FFA when measured and include palmitate (16:0, ~23% of the total lipids, ~585 μmol/L controls *vs*. T2D ~24% of the total lipids, ~801 μmol/L), oleate (18:1, ~19% of the total lipids, ~450 μmol/L controls *vs*. T2D ~21% of the total lipids, ~688 μmol/L), stearate (18:0, ~7% of the total lipids, ~159 μmol/L controls *vs*. T2D ~7% of the total lipids, ~227 μmol/L), linoleic acid (18:2, ~31% of the total lipids, ~744 μmol/L controls *vs*. T2D ~31% of the total lipids, ~1036 μmol/L) and arachidonic acid (20:4, ~9% of the total lipids, ~222 μmol/L controls *vs*. T2D ~8% of the total lipids, ~284 μmol/L) (43, 44, 61–63). This data suggests that there is about a 40% increase in total and the five main FFAs found in plasma in T2D patients, which is consistent with previous publications (66, 68, 101–103). These values also give us a good starting point for planning chronic *in vitro* studies with isolated islets. For example, it suggests control islets should be treated with FFA, as discussed above, as well as the high-fat treated islets, but the difference between control and high-fat treated islets should be around 40%. This idea goes against what is usually done where control islets are typically treated with FFA-free BSA, which results in a non-physiological concentration of FFA, but also, the FFA-free BSA can pull FFA and other hydrophobic molecules from islets, complicating the interpretation of the results. Another problem with chronically treating islets with FFA is that we cannot perform long-term studies. What is typically done is to treat islets *in vitro* with high levels of FFA for 48–72 hrs to mimic what is happening in type 2 diabetes. However, type 2 diabetic islets *in vivo* are exposed to lower levels of elevated FFA over years and not days. Thus, *in vitro* comparisons to what is happening *in vivo* are challenging to interpret and may represent two different mechanisms in the development of lipotoxicity.

In addition to plasma FFA, there are changes in some of the other lipids found in non-fasted or overnight-fasted human plasma in type 2 diabetes. For example, the average TG levels are 1.13 µmol/Lin healthy matched control patients *vs*. 1.78 µmol/L in T2Ds. Total cholesterol is 4.76 µmol/L in healthy patients *vs*. 5.05 µmol/L in T2Ds. HDL-cholesterol is 1.34 µmol/L in healthy patients *vs*. 1.19 µmol/L in T2Ds. LDL-cholesterol is 2.97 µmol/L in healthy patients *vs*. 3.21 µmol/L in T2Ds. The TG, total cholesterol, HDL-cholesterol and LDL-cholesterol levels described were averaged from several papers (43, 44, 63–68). Although these differences are not large, as a whole, T2Ds have abnormal plasma lipid levels, which is consistent with previously published studies. The use of lipids should be comparable and carefully considered when planning *in vitro* islet studies so that they more closely match what is going on *in vivo*.

Most lipid studies have focused on non-fasted or short-term fasting plasma lipid levels, and the role of lipids in the development of T2Ds may be more complex than what is seen in the fasting state. T2Ds have been found to have higher intakes of total and saturated fat than healthy controls (91, 104), although this is not always the case in all studies (92, 105, 106). In addition, low-fat diets may not be as beneficial for avoiding T2Ds as researchers hoped (90, 92, 105, 107–109). There have also been limited studies looking at what happens to lipids postprandially in T2Ds, partly due to the complexity of planning such studies. Postprandial lipid absorption, transport and metabolism may be altered in T2Ds, and more clinical studies are needed to assess this possibility.

When treating islets with FFA, one should consider the *in vivo* changes in lipids. Researchers should ask whether there is a need to balance the total FFA levels when assessing the effects of a single FFA, such as palmitate. For example, if we only use palmitate at its physiological concentration discussed above and conjugate it to albumin, do we use a lower albumin concentration to be sure we have an appropriate level of unbound palmitate, or do we conjugate palmitate to a physiological concentration of albumin and use other lipid(s) to balance the total FFA in the preparation? Using a lower albumin concentration or adding another lipid will complicate the interpretation of the results. Also, what controls should be used for these experiments? The answer to these questions is not straightforward since untreated albumin may have lipids and other impurities, and using FFA-free albumin may pull endogenous FFAs and other lipid-soluble metabolites out of the cells you are trying to treat. FFA-free albumin will also impact experiments using lipid-soluble drugs since albumin can bind these as well (20).

## FFA, albumin and isolated islet studies

The difficulty in comparing many *in vitro* islet studies is that the type and final concentration of BSA is variable between studies and is typically between 0.1-1% (a 1% BSA solution is equivalent to 1 g/dl or 151 µM BSA solution). Interestingly, the albumin concentration of *in vivo* plasma is about 3.5–5 g/dl (or 3.5-5%, 530-758 µM), which is far higher than that used for *in vitro* islet studies (110). The interstitial concentration of albumin in the space around islet cells and whether interstitial albumin plays an important role in the exposure of islet cells to FFAs is unknown. The estimated interstitial albumin concentration in the space around cells is challenging to determine experimentally. In one study, it was estimated to be 0.73 g/dl (111 µM) in adipose and 1.3 g/dl (197 µM) in skeletal muscle (111). These studies suggest a reasonable estimate for the interstitial albumin concentration around islets is about 151 µM (or 1%); however, this should be determined experimentally.

Another question is whether interstitial albumin is more important than the plasma concentration of albumin in controlling the exposure of islets to unbound FFAs. The delivery of FFAs to islets is challenging to estimate because they are not solely dependent on total circulating FFAs and albumin but also on local delivery of FFA. Islet lipoprotein lipase on or near islets can increase local FFA levels from TG stores in chylomicrons and VLDLs. Also, FFAs released by islets from internal stores may play a role in local FFA delivery to islets (**Figure 1**) (112, 113). The local unbound concentration of FFA is important in controlling cellular uptake (23), and only the FFAs that remain unbound can potentiate glucose-stimulated insulin secretion (GSIS) (10). Therefore, understanding the above questions is essential in effectively planning *in vitro* islet experiments.

**Figure 2:**
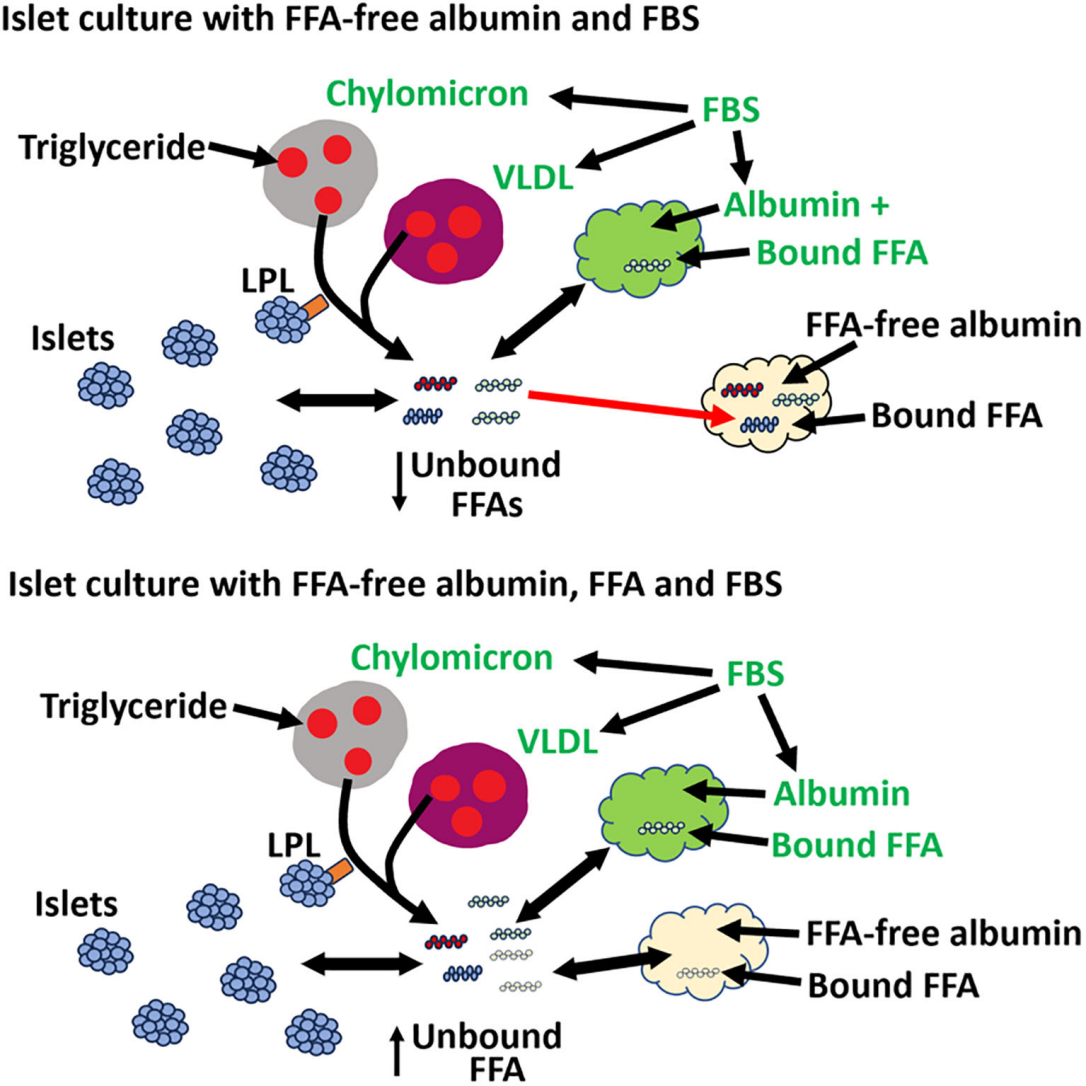
The concentration of FFAs in islet culture media depends on whether FFA-free albumin is used, if the FFAs are bound or unbound, and if FBS is included in the media. Top: Control islet experiments are typically incubated with FFA-free albumin (yellow). For long-term islet studies, fetal bovine serum (FBS) is also included in the culture media. FBS contains albumin (green) with bound FFAs (green FFA), chylomicrons (grey) and VLDLs (purple). Lipoprotein lipase (LPL, orange) on islets can release FFAs (red FFA) from TG stored in chylomicrons and VLDLs. FFA can also be released from internal stores of islets (blue FFA). Overall, there are two sources of unbound FFAs in control experiments: FFAs from islets and FFAs from FBS (chylomicrons, VLDL, and albumin). These unbound FFAs can bind to FFA-free albumin (indicated by red arrow) in islet culture media, which will reduce the unbound concentration of FFAs and potentially pull FFA (blue FFA) from islets and FBS (green FFA and red FFA). Bottom: FFA-treated islet experiments are typically incubated with FFA-free albumin pre-conjugated with FFA (yellow FFA) and sometimes FBS. In this experimental setup, there are three possible sources of FFAs that contribute to the unbound FFA concentration. These include 1) FFAs from albumin, Chylomicrons, and VLDL from FBS, 2) FFAs from islets, and 3) the FFA prepared with FFA-free albumin pre-conjugated with FFAs. The combination of all three sources of unbound FFA may increase the final unbound FFA above what was wanted experimentally.

After complexing albumin and FFAs, one must also consider which media is used to treat cells (**Figure 2**). For example, it is common to use fetal bovine serum (FBS) to culture islets that are being treated with chronic high fat. FBS has a concentration of albumin around 2-4% (on average 2.5 g/100 ml) and has FFAs, cholesterol, chylomicrons, VLDL, LDLs, HDLs and other impurities (11, 114). For example, a typical value of 10% FBS in the islet culture media will increase the albumin concentration by ~0.25%. FBS also has around 190 µM of total FFAs, which, when diluted to 10% FBS, will add 19 µmol/L of FFAs to the experimental media (114). Islet cells also express lipoprotein lipase that can release FFA from TGs stored in chylomicrons and VLDL found in FBS (112, 115, 116). These variables must be carefully controlled for when planning *in vitro* islet studies.

Overall, interpreting lipid-based experiments in islets and comparing different studies is complicated by a number of variables. In a scan of several lipid studies in islets, there is sometimes incomplete information on the methods of lipid preparation, storage time, or the molar ratio of FFA: albumin (117–121). For future studies, it is essential to report the method of preparation, whether a concentrated stock of FFA was used, the time from preparation to use in experiments, was FBS used, what media/buffer was used for the experiments, what controls are used, FFA/albumin molar ratio, and unbound FFA concentrations should be measured for *in vitro* experiments (**Figure 2**). There is also a need to understand better how lipids are delivered to islet cells across blood vessels and through the interstitial space to better plan *in vitro* islet experiments. Since albumin plays such an important role in solubilizing FFA, it is important to determine the interstitial albumin concentrations near islets to allow us to create a more accurate physiological model of what is happening *in vivo*.

## FFA transport into cells

Transport of FFA from blood vessel capillaries into cells is not trivial. The movement of FFA from blood plasma to cells is limited by dissociation of FFA from albumin, transport across or in between capillary endothelial cells, binding and release from interstitial albumin, binding of FFA to the outer leaflet of the cell plasma membrane, transport across the plasma membrane into the inner leaflet of the plasma membrane and final binding to intracellular fatty acid binding proteins (FABP) and lipid activation (17) (**Figure 1**). FABP has a lower binding affinity as compared to albumin, and its expression changes depending on the nutrition state of the cell (122, 123). To complicate matters further, the expression of lipoprotein lipase on capillary endothelial cells (124) or on islet β-cells (112, 115, 116) has been shown to release FFA from TG in chylomicrons and VLDL, which can increase the local concentration of FFA around islet cells *in vivo*. The expression of lipoprotein lipases is also affected by the nutritional state of islet cells (125). For *in vitro* islet studies, some of these barriers are bypassed, but new obstacles are introduced. For example, transport of FFA to the core of an islet is limited without blood transport of FFA via islet core capillaries. Also, the expression and activity of lipoprotein lipase in islets should be considered when performing *in vitro* islet lipid studies.

## FFA activation and metabolism in cells

**Figure 3:**
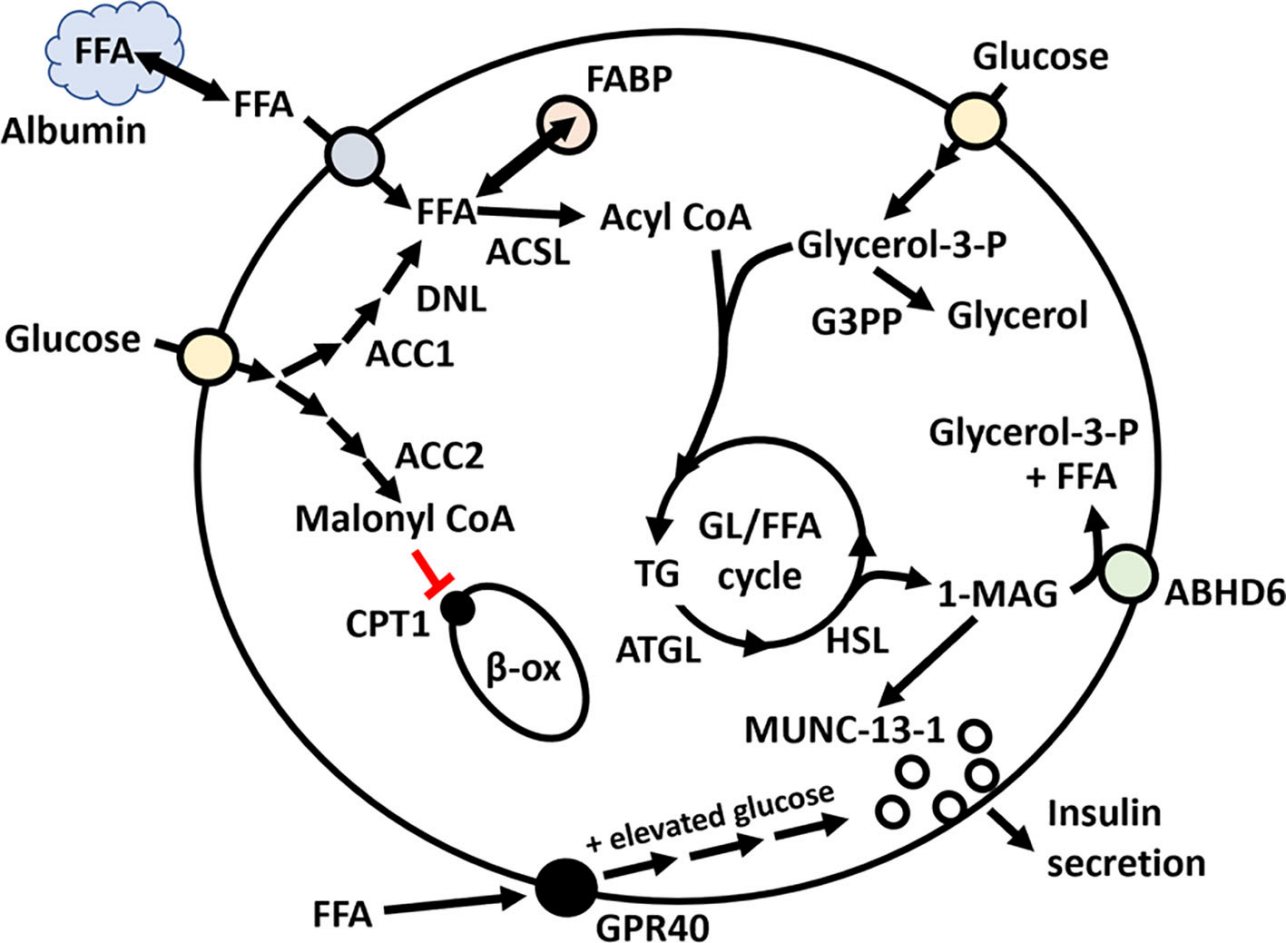
The role of FFAs in the potentiation of glucose-stimulated insulin secretion from islet β-cells. FFA can either activate GPR40 or be transported into β-cells, followed by activation by ACSL to acyl-CoA. GPR40 requires elevated glucose levels to stimulate insulin secretion. In addition to external sources of FFA, FFA can be generated from glucose, involving the *de novo* lipogenesis (DNL) pathway. Glucose can also be converted to malonyl-CoA, which can inhibit β-oxidation in mitochondria. Acyl-CoA and glycerol-3-phosphate can enter the GL/FFA cycle, which cycles between TG synthesis and lipolysis, generating 1-monoacylglycerol (1-MAG). 1-MAG can interact with MUNC13-1 and promote insulin secretion. 1-MAG is hydrolyzed by ABHD6 to FFA and glycerol-3-phosphate.

Once inside islet cells, FFA is important in regulating insulin secretion (**Figure 3**). Most lipid metabolism pathways require lipids to be first activated by thioesterification to acyl-CoA. Exogenous and endogenous FFA are trapped in cells by converting them into acyl-CoAs (126). Long-chain acyl-CoA synthetases (ACSL) and FA transport proteins (FATP) have acyl-CoA synthase activity and enhance FA uptake (127). FAs of 14–26 carbons are activated by one of 13 long-chain or very-long-chain acyl-CoA synthetases (ACSL, ACSVL/FATP, ACS bubblegum (ACSBg)) (128). Once activated, acyl-CoA can be metabolized by one of six major enzyme families: elongases and desaturases (129), dehydrogenases (130, 131), acyl-CoA thioesterases (132, 133), carnitine palmitoyltransferases (CPT) (134), and lipid and protein acyltransferases (135). In addition, acyl-CoA can be used for cell signaling, vesicle fusion, and protein acylation at the plasma membrane, endoplasmic reticulum, and Golgi (136–145). Acyl-CoA are not freely moving in cells but are directed into specific pathways, and their metabolism is highly controlled, and the movement of the acyl-CoAs themselves seems to be compartmentalized, but how is unknown.

There are five mammalian isoforms of ACSL (ACSL1, 3-6), which can be further subdivided into two subfamilies depending on their substrates and amino acid sequence (146–148). Although all five ACSLs use saturated and unsaturated fatty acids of chain lengths of 8–22 carbons, ACSL1 has a marked preference for oleate and linoleate (147–149). The preferred substrates of ACSL3 are myristate, palmitate, arachidonate, and eicosapentaenoate (128, 150–152). ACSL4 has a marked preference for arachidonic acid (20:4) and eicosapentaenoic acid (20:5) (150–154). ACSL5 preferred substrates are palmitate, palmitoleate, oleate, and linoleate (147, 148, 154). ACSL6 is reported to have an equal preference for saturated and polyunsaturated FA with a backbone of C16-C20 (147, 148, 155). ACSLs play a role in directing fatty acids to various lipid metabolic pathways, including complex lipid synthesis, lipid storage or lipid β-oxidation (156). It has been shown that ACSL1 may shunt acyl-CoAs towards TG synthesis, whereas ACSL5 may shunt acyl-CoAs towards mitochondrial β-oxidation. ACSL4 may provide acyl-CoA to peroxisomes for lipid synthesis and oxidation. However, more work is needed to examine the role of ACSLs in shunting acyl-CoAs to various pathways (156). In β-cells, ACSL3 and ACSL4 are expressed in human and rat islets and are required for optimal glucose-stimulated insulin secretion (157, 158). ACSL3 and ACSL4 are concentrated on insulin granules and less so in mitochondria.

Support for the compartmentalization of ACSL proteins comes from several studies. Compartmentalized production of acyl-CoA is supported by studies showing that ACSL1 knockout in the liver, which is equally distributed on the ER and the mitochondrial outer membrane and accounts for only 50% of the total ACSL activity, leads to minimal loss of TG synthesis and β-oxidation (159, 160). Whereas the loss of ACSL1 in skeletal muscle, brown fat and cardiac tissue, where ACSL1 accounts for 90% of total activity, leads to a decrease in β-oxidation, but the remaining ACSL isoforms are sufficient for normal TG and membrane phospholipid biosynthesis (161–163). However, the problem with most studies looking at acyl-CoA partitioning by ACSL isoforms is that these studies have been done using subcellular fractionation that can result in contamination from other cellular compartments (164). Using confocal imaging is a better choice; however, these studies require fixed cells and highly specific antibodies. Overexpression studies have also been performed with tagged ACSL proteins with fluorescent proteins; however, abnormally high protein levels complicate these studies, and these studies may lead to ER retention and not reflect the true endogenous protein localization (165–167). Also, localization may depend on the type of cell or the cell’s physiological state and how the cells have been treated. For example, exogenous FA causes tagged ACSL3 to move from the ER to newly forming lipid droplets (168), and knockdown of ACSL3 reduces *de novo* lipogenesis (DNL) (169, 170). ACSL1 and ACSL5 have been localized to mitochondria and ER and likely play a role in shunting lipids towards β-oxidation and TAG synthesis (171–173). Gaining a better understanding of the role of lipid transport and activation in β-cells and the changes that occur during different nutritional states is essential to interpreting the effects of treating islets with acute and chronic elevation of lipids.

## Acute effects of FFA on insulin secretion

Lipids are required for glucose-stimulated insulin release *in vivo* (60) and *ex vivo* (8), and if islets are deprived of FFA, their response to glucose is impaired (174). There are three possible sources of islet FFAs: 1) exogenously derived FFAs (dietary and circulating), 2) endogenously synthesized FFAs, and 3) release from internal stores. FFA can potentiate glucose-stimulated insulin secretion by a receptor-based mechanism involving the G-protein-coupled receptor 40 (GPR40) and through lipid metabolism that generates insulin secretory signalling molecules (5, 175, 176). Several lipid metabolism pathways have been shown to play a role in regulating insulin secretion, including the ATP citrate lyase (ACL)/acetyl-CoA carboxylase (ACC)/malonyl-CoA/carnitine palmitoyltransferase-1 (CPT-1) axis and the glycerolipid (GL)/FFA cycling pathway (177) (**Figure 3**).

FFA activation of GPR40 (or FFAR1) has been shown to potentiate glucose-stimulated insulin secretion at high glucose but not at low glucose concentrations in clonal cell lines (INS-1 and MIN6 cells) and mouse and human islets (176, 178–181). Using GPR40 knockout mice, it has been suggested that about 50% of the insulin secretion response to FFA is due to activation of GPR40 (182). GPR40 activation leads to the activation of phospholipase C via the Gaq/11 subunit and an increase in intracellular calcium concentrations (178). It has also been suggested that GPR40 may be coupled to the Gαs subunit, activating adenylyl cyclase and mediating changes in ion channel activities (183).

Deletion of GPR40 in mice does not affect *in vivo* glucose metabolism under normal physiological conditions (175, 182, 184). Feeding GPR40 knockout mice a high-fat diet leads to the development of fasting hyperglycemia and lower insulin secretion (176). In addition to GPR40, GPR120 (FFAR4) has also been suggested to regulate insulin secretion. Like GPR40, GPR120 can potentiate islet glucose-stimulated insulin secretion (185). However, GPR120 knockout has been shown to have normal β-cell function (186, 187). Using GPR40 and GPR120 knockout mice and double knockout mice, it has been demonstrated that activation of both GPR120 and GPR40 enhances insulin secretion *ex vivo*. However, the combined deletion of these two receptors only minimally affects glucose homeostasis *in vivo* in mice (188).

Although studies in mice do not always show an essential role for GPR40 in regulating glucose homeostasis, this may not be the case in humans. Since GPR40’s effects on insulin are glucose-dependent, it suggests that it could be a target for treating type 2 diabetes. The GPR40 agonist, TAK-875 (Fasiglifam), has been shown to reduce fasting and postprandial blood glucose levels and HbA1c in clinical trials (189). However, phase III trials were terminated due to safety concerns. Although unsuccessful, these clinical trials have demonstrated the potential for targeting GPR40 in treating type 2 diabetes, and newer compounds with fewer side effects are being developed (189, 190).

Lipid-regulated insulin secretion in β-cells involves the GL/FFA cycling pathway. This pathway involves both glucose-driven DNL production of malonyl CoA, FFA, and acyl-CoA and the release of lipids from internal stores (4, 191, 192). β-cells are capable of lipogenesis and contain enzymes required for lipid synthesis, including pyruvate carboxylase (PC) (193, 194), fatty acid synthase (FASN) (194, 195) and acetyl-CoA carboxylase (ACC) (195). Glucose-driven DNL in β-cells has been proposed to generate metabolic signalling molecules important for regulating insulin exocytosis (4). A key step in the DNL pathway is the export of mitochondrial citrate into the cytosol, which is then converted to acetyl CoA and oxaloacetate by citrate lyase (CL) (196). Acetyl CoA can be converted to malonyl-CoA by ACC (5, 192, 197). The generated malonyl CoA can inhibit carnitine palmitoyltransferase 1 (CPT-1) and β-oxidation, which is an essential step in regulating insulin release (198). FASN synthesizes long-chain fatty acids by using acetyl-CoA as a primer, malonyl-CoA, and NADPH as a reducing equivalent. FASN predominately produces the 16-carbon non-esterified fatty acid palmitate, which can be modified into other types of FFAs. Interestingly, FFAs cannot stimulate insulin secretion in the absence of elevated glucose (192, 199). FFA activation to acyl-CoA by ACSL is also an essential part of the ability of lipids to potentiate glucose-stimulated insulin secretion (12, 157, 158, 192).

The lipolysis arm of the GL/FFA cycle consists of the breakdown of triglycerides and phospholipids to give rise to glycerol and FFA (192). Four GL/FFA cycle enzymes, hormone-sensitive lipase (HSL), adipose triglyceride lipase (ATGL), glycerol 3-phosphate phosphatase (G3PP) and α/-β-hydrolase domain 6 (ABHD6), have been shown to play a vital role in β-cell insulin secretion (200–203). G3PP hydrolyses glucose-derived glycerol-3-phosphate (204), and ABHD6 controls the last step of lipolysis by hydrolyzing 1-monoacylglycerol (1-MAG) (202). G3PP controls the availability of glucose-derived glycerol 3-phosphate, the precursor for generating triglycerides (204) whereas ABHD6 hydrolyzes 1-MAG, which has been suggested to be a key signalling molecule involved in regulating glucose-stimulated insulin release by activating Munc13-1, an exocytosis-facilitating protein (202). Since 2-arachidonoylglycerol (2-AG) is the main 1-MAG species hydrolyzed by ABHD6, it suggests that 2-AG can activate Munc13–1 to promote insulin secretion. These studies show that ABHD6 plays an essential role in regulating FFA-stimulated insulin secretion. Glucose can increase lipolysis from lipid droplets in human islets, and this process is defective in type 2 diabetic islets (203). Both ATGL and HSL play important roles in the mobilization of lipid droplets from β-cells, and their loss can lead to defective insulin secretion (200, 203, 205).

Another critical step in regulating lipid metabolism in β-cells is inhibiting lipid metabolism via β-oxidation. Several studies have shown that promoting β-oxidation either by overexpressing CPT1 or expressing a malonyl CoA insensitive CPT1 leads to inhibition of insulin secretion and suggests that malonyl CoA, ACC, and CPT1 are essential players in regulating insulin secretion (4, 198, 206). Although somewhat controversial, β-cells express both ACC1 and ACC2. At the single-cell level, human β-cell ACC1 mRNA is expressed about 21-fold higher than ACC2, and ACC1 is expressed about 13-fold higher than ACC2 in mouse β-cells (207). Additional studies have also shown that ACC2 is expressed in mice, rat islets and clonal β-cells (832/13 cells) (197, 208, 209) and human β-cells (207, 210, 211). We have also published three papers (208, 209, 212) showing that ACC2 is expressed in mice, rat islets and clonal β-cells (832/13 cells). The loss of PHD3 in β-cells may increase the risk of developing diabetes and may be related to the loss of PHD3-mediated proline hydroxylation of ACC2 (208, 209, 213, 214).

Although ACC2 expression in β-cells has been debated, a number of studies have shown that ACC2 is required for the inhibition of β-oxidation of lipids and plays a role in regulating insulin secretion (4, 57, 198, 206, 207, 210, 211, 215–224). The protein expression of ACC2 has been shown in clonal β-cells and islets in several papers (225–229), although a few papers have suggested ACC2 is not expressed at the protein level (195, 230). ACC2 is embedded in the mitochondrial outer membrane and regulates β-oxidation of fatty acids by generating malonyl CoA, inhibiting CPT1 and lipid transport into mitochondria (231). The inhibition of β-oxidation requires malonyl CoA generated by ACC2, whereas the malonyl CoA generated by ACC1 is used for DNL (reviewed in (232)). Strong support for the role of ACC2 in the regulation of CPT1 and β-oxidation in β-cells was shown in several papers (197, 233). These papers showed that the knockdown of ACC1 in clonal cells and β-cell-specific ACC1 knockout islets inhibits DNL, but these cells maintain the ability of glucose to inhibit β-oxidation, which is consistent with a key role for ACC1 in controlling DNL and ACC2 regulating β-oxidation in β-cells. Defects in the ability to regulate lipid β-oxidation in β-cells are known to lead to an inhibition of insulin secretion (209, 215).

## Chronic effects of FFA on β-cell function

*In vitro* islet studies looking at the effects of chronic lipid treatment use different types of fatty acids and forms at various concentrations (e.g., palmitate or palmitic acid, oleate or oleic acid). In studies investigating lipotoxicity, variable and sometimes high non-physiologically molar ratios of FFA/BSA are used (46, 47, 49, 51, 53–56, 234, 235). If we compare the molar ratios used *in vitro* to what is seen in people with T2Ds, non-diabetic plasma FFA levels are typically between 278-580 µmol/L, and T2Ds are between 370-830 µmole/l (62, 64, 65, 68, 103). Albumin concentrations in human plasma are between 3.5–5 g/dl (or 3.5-5%, 530-758 µM) (110). Thus, using an albumin concentration of 650 µM and the highest measured plasma FFA levels, the ratio of FFA: albumin for non-diabetics is ~0.89 and T2Ds ~1.27. The molar ratio of FFAs: albumin provides information on the unbound concentration of FFAs. To compare studies, it is important to know the method of lipid preparation, BSA concentration and FFA/albumin molar ratio, unfortunately, this information is not always clearly stated or provided (117–121). In well-controlled *in vitro* islet studies that provide detailed methods of lipid preparation, a typical FFA: albumin ratio is between 3:1 and 6:1 (36, 37, 56, 234–236). One of the common explanations for using higher molar ratios is to overcome the experimental time barriers since the development of type 2 diabetes takes years *vs*. our ability to culture islets for long periods. However, changing the FFA: albumin molar ratio and the incubation time may significantly impact the biological effects and mechanism of impairment of islet function (36, 236). It has also been suggested that molar ratios greater than 6:1 should be avoided as they are unphysiological and may exceed FFA solubility, which may produce artifacts (36, 237).

The method of conjugating lipids to BSA, whether stock solutions are used, and the length of storage before use can also complicate matters further. For example, treating INS-1E cells with 0.5 mM palmitate conjugated with 0.75% FFA-free commercially purchased BSA solution (FFA/albumin molar ratio of 4.4) for 16 hours resulted in a similar level of apoptosis as seen with a solution made with 1% charcoal-absorbed BSA (FFA/albumin molar ratio of 3.3) (36). Interestingly, if FFAs are precomplexed with FFA-free BSA at a higher concentration and then diluted to a similar degree as a solution made without diluting the FFAs, led to less apoptosis, suggesting stock solution storage affects FFA/BSA solution (36). This may be related to some of the FFA forming aggregates in the pre-complexed FFA/BSA solution, which would lower the available unbound FFAs (36). Also, when oleate is prepared and used at a similar final concentration to palmitate, it leads to a comparable level of apoptosis (36). However, when unbound FFA was set at 24 nM for both palmitate and oleate, palmitate led to more apoptosis than oleate (36). These studies suggest that the type of FFA-free BSA and preparation method can significantly impact the results when treated with lipids.

## Conclusions

Overall, lipids play a key role in regulating β-cell function; however, understanding how they regulate insulin secretion is complicated by a number of factors. Variability in *in vitro* islet experimental methods include the type of lipid, the form of the lipid, concentration and type BSA, method of preparation, delivery to cells, and the concentration of unbound FFA. Additionally, how islet lipid experiments are performed should be carefully planned to use a more physiological approach to assessing the role of FFA on insulin secretion. For example, controls should have physiological levels of FFA, and the molar ratio of FFA/BSA should be less than 3:1 since FFA levels don’t change more than that in most disease states, including type 2 diabetes. It is well accepted that FFA plays a role in regulating insulin secretion in response to a meal, in the development of type 2 diabetes, and chronic exposure to FFA can result in β-cell dysfunction. However, how FFA are delivered to islets is incompletely understood. One key source of local FFA delivery to islets *in vivo* may be lipoprotein lipase release of FFA from chylomicrons and VLDL. Overall, to allow for comparison of different islet lipid studies, it is recommended that isolated islet studies using FFA should:

1. Report the type, source and order information of albumin and FFA.
2. Report the method of lipid preparation and the amount of time the lipids are stored.
3. Consider the potential effects of using different albumin concentrations and FFA-free BSA.
4. Report the FBS concentration in the islet culture media and consider the potential release of FFA from chylomicrons and VLDLs in FBS from islet lipoprotein lipases.
5. Measure the final concentration of FFA and unbound concentration of FFA.
6. Report the final concentration of albumin, FFAs, unbound FFA concentration, and the molar ratio of FFA: albumin.
7. Avoid molar ratios of FFA: albumin greater than 3:1.
